# Composition of Algerian Propolis, Plant Origin, and Its Antiangiogenic Activity In Vitro

**DOI:** 10.3390/molecules26216510

**Published:** 2021-10-28

**Authors:** Takahiro Hosoya, Ikumi Tsuchiya, Toshiro Ohta, Mokhtar Benhanifia, Shigenori Kumazawa

**Affiliations:** 1 Department of Food and Nutritional Sciences, University of Shizuoka, 52-1 Yada, Suruga-ku, Shizuoka 422-8526, Japan; hosoya@toyo.jp (T.H.); tsuchiya2021@u-shizuoka-ken.ac.jp (I.T.); ohtat@u-shizuoka-ken.ac.jp (T.O.); 2 Department of Nutrition and Health Sciences, Toyo University, 1-1-1, Izumino, Itakura-machi, Ora-gun, Gunma 374-0193, Japan; 3 Department of Agricultural Science, Faculty of Natural and Life Sciences, University Mustapha Stambouli of Mascara, Mascara 29000, Algeria; benhanifia@gmail.com

**Keywords:** propolis, antiangiogenic, ferulenol, plant origin, Algeria

## Abstract

The antiangiogenic activity of the ethanol extract of propolis collected from different regions in western Algeria was investigated using in vitro human umbilical vein endothelial cells (HUVECs). The ethanol extract with the strongest activity, i.e., Algerian propolis 1 (EEPA1), inhibited the formation of capillary networks in a dose-dependent manner (6.25–50 μg/mL) within 12 h and induced cell fragmentation of HUVECs at 50 μg/mL after treatment for 24 h. To identify the active compounds in EEAP1, a high-performance liquid chromatography (HPLC) analysis was performed, revealing that EEAP1 contains two major compounds. Both compounds were isolated by repeated column chromatography and identified as ω-hydroxyferulenol (**1**) and ferulenol (**2**), which have a coumarin structure conjugated with a farnesyl group according to NMR, high-resolution electrospray ionization mass spectroscopy, and chemical modification. Compounds **1** and **2** inhibited the tube-forming activity of HUVECs, especially **2**, which exhibited a stronger antiangiogenic effect even at a low concentration of 3.31 μg/mL. Moreover, **2** suppressed the elongation and induced cell fragmentation at the same dose. The molecular changes in tube-forming HUVECs induced by **2** were found to be related to the activation of the caspase signals. To confirm the plant origin of propolis, an HPLC comparative analysis of the ethanol extracts of some plants near beekeeping areas and that of Algerian propolis (EEAP1) was performed, and similar chromatographic patterns were observed. This result suggests that the plant origin of this Algerian propolis is the resin of *Ferula communis*.

## 1. Introduction

Propolis is a yellow-green to dark brown resinous hive product that is collected and processed by honeybees from plant buds, exudates, and other botanical sources. It is believed to serve as a protective barrier for the beehive. The main constituents of propolis are beeswax and plant resins. Bees collect these substances on their hind legs and carry them to the hive to seal holes and build structures. It has been also recognized since ancient times that propolis protects the hive against pathogenic microorganisms [1,2,3], which is an essential characteristic of propolis. Hence, propolis has been used as a traditional folk medicine worldwide [4,5,6] because of its antioxidant [7,8], antibacterial [9,10], anti-inflammatory [11,12], and anticancer activities [13,14]. Accordingly, propolis has gained popularity as an alternative medicine for the prevention and treatment of inflammation, diabetes, heart disease, and cancer [8,14,15].

Studies on the chemical composition of propolis have demonstrated its compositional variability, which largely depends on the source plant(s) [16,17]. The most extended type worldwide is poplar-type propolis derived from poplar buds [18]. However, green propolis, which stems from the buds of *Baccharis dracunculifolia* DC, is the most common type in the southeastern area of Brazil [19] and red propolis, derived from *Dalbergia ecastaphyllum* (L.) Taub., is collected in the northeastern part of Brazil [20,21]. The source plants of various propolis have been identified by combining bee behavior observation and chemical analysis of both plant resins and propolis. This has allowed for the identification of source plants in Okinawa, Japan (*Macaranga tanarius* (L.) Müll. Arg.), and in Jeju Island, Korea (*Angelica keiskei* Ito) [22,23]. Nevertheless, other types of propolis worldwide likely remain undiscovered.

The chemical composition of propolis is qualitatively and quantitatively variable depending on the vegetation in the area from which it is collected [16,18,24]. For instance, poplar-type propolis contains typical poplar bud phenolics such as flavonoid aglycones and phenolic acids (and their esters) [16,18]. By contrast, the main compounds found in Brazilian green propolis are prenylated derivatives of *p*-coumaric acid, diterpenes, lignans, and flavonoids [16].

Our main goals were to discover a new type of propolis and to clarify its biological activity. This study describes the components and biological activity, particularly the antiangiogenic activity, of propolis samples collected from different locations in western Algeria. Although several pharmaceutical studies on the antioxidant, antimicrobial, and anticancer activities of Algerian propolis have been conducted [25,26,27], to the best of our knowledge, the antiangiogenic activity of Algerian propolis has not been reported yet. In our previous paper, on the basis of in vitro and in vivo angiogenesis models, we reported that Brazilian and Okinawan propolis possess antiangiogenic activities [13,28]. Angiogenesis is defined as the process of the formation of new blood vessels from preexisting ones and antiangiogenic activity may be useful in the treatment and prevention of cancer progression [29,30]. In this study, we report the chemical analysis, antiangiogenic activity in vitro, and probable botanical source of Algerian propolis.

## 2. Results

### 2.1. Investigation of the Anti-Tube-Forming Activity of Algerian Propolis

**Figure 1:**
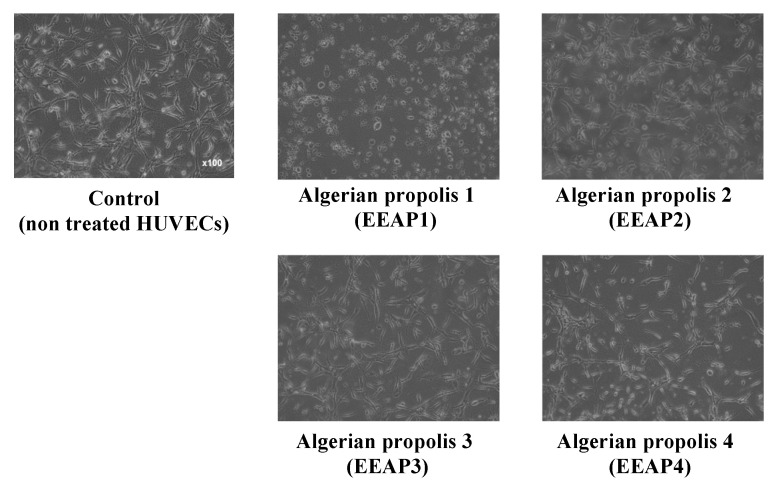
Inhibitory effects of ethanol extracts of Algerian propolis 1–4 (EEAP1–4) on the tube formation of HUVECs. The cells were treated with 50 μg/mL of EEAPs and observed after 12 h. EEAP1 showed the strongest antiangiogenic activity, completely inhibiting the formation of capillary networks and inducing cell fragmentation.

**Figure 2:**
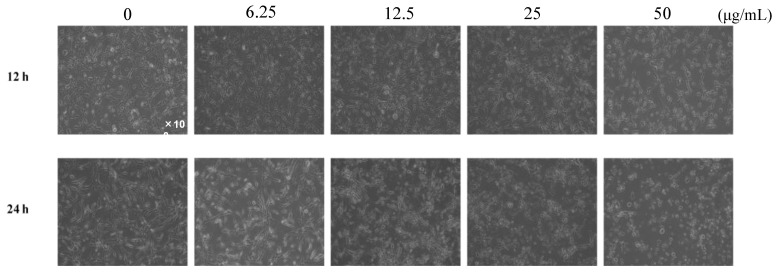
Inhibitory effects of ethanol extracts of EEAP1 on the tube formation of HUVECs. EEAP1 at 50 μg/mL inhibited the elongation of HUVECs and the formation of capillary networks after 12 h. After 24 h treatment, EEAP1 dose-dependently inhibited the formation of capillary networks.

The antiangiogenic effect of the ethanol extract of four kinds of propolis (EEAP1, EEAP2, EEAP3, and EEAP4) collected in different locations of western Algeria (Table 1) was investigated by performing a tube formation assay in vitro. As shown in Figure 1, EEAP1 had the strongest antiangiogenic activity, completely inhibiting tube formation and inducing cell fragmentation, which is a morphological hallmark of apoptosis, of human umbilical vein endothelial cells (HUVECs) at 50 μg/mL after 12 h of treatment. To investigate the antiangiogenic activity of EEAP1 in detail, tube-forming HUVECs were treated with several doses of EEPA1 for 12 and 24 h. Treatment with EEAP1 at 50 μg/mL inhibited the elongation of HUVECs and the formation of capillary networks after 12 h. Furthermore, EEAP1 treatment for 24 h inhibited the formation of capillary networks in a dose-dependent manner and EEAP1 at 50 μg/mL induced cell fragmentation of HUVECs (Figure 2).

### 2.2. Identification of the Main Compounds in EEAP1

**Figure 3:**
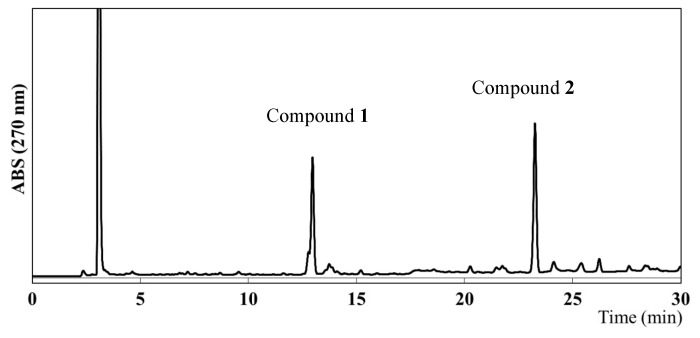
HPLC chromatogram of the ethanol extract of Algerian propolis 1 (EEAP1).

To identify the compounds responsible for the antiangiogenic activity in EEAP1, a high-performance liquid chromatography (HPLC) analysis was performed, which revealed the presence of two main compounds, as shown in Figure 3. Column chromatography and preparative HPLC (see the Experimental Section for details) led to the isolation of two compounds (**1** and **2**). However, the HPLC chromatograms of the isolated compounds **1** and **2** showed two peaks each. Assuming that the isolated compounds might form two derivatives in hydrogen donor solvents, methylation with trimethylsilyldiazomethane (TMSCHN_2_) was performed on the crude fraction before the isolation procedure and the structures of **1** and **2** were determined using their methyl derivatives. The methylated fraction was subjected to preparative HPLC to provide four peaks with retention times of 14.5, 15.8, 24.8, and 27.0 min, which were assigned as compounds **3**, **5**, **4**, and **6**, respectively. These compounds were analyzed via high-resolution electrospray ionization mass spectroscopy (HR-ESIMS) as well as via one-dimensional (1D) and two-dimensional (2D) nuclear magnetic resonance (NMR) spectroscopy.

**Figure 4:**
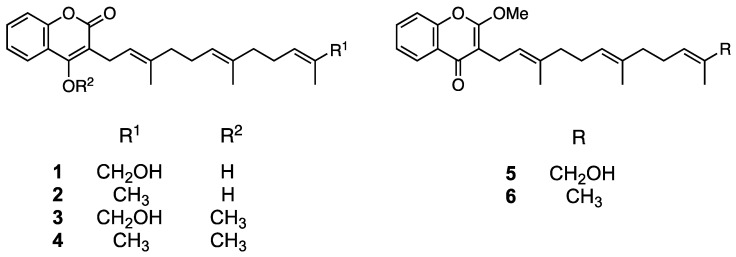
Chemical structures of **1** (ω-hydroxyferulenol), **2** (ferulenol), and their methylated compounds (**3**–**6**).

Compound **3** was obtained as an amorphous solid and its molecular formula was determined to be C_25_H_32_O_4_ by HR-ESIMS (*m/z* 419.2194 [M+Na]^+^ calculated for C_25_H_32_O_4_Na, 419.2198; Figure S3). The ^1^H NMR spectrum of **3** in acetone-*d*_6_ showed signals corresponding to aromatic protons (δ 7.50–8.00), a methoxyl group (δ 4.06), and three methyl groups (δ 1.50–1.70; Figure S4). The ^13^C NMR spectrum showed 25 signals attributable to a methoxy group (δ 62.4), aromatic or olefinic carbons (δ 102.4–136.4), two aromatic/olefinic carbons conjugated with a hydroxyl group (δ 153.5 and 163.4), and a carbonyl carbon (δ 163.9; Figure S5). The 2D NMR spectra of **3** revealed the presence of a coumarin structure conjugated with a farnesyl group (Figures S6 and S7). Hence, a correlation between methoxyl protons (δ 4.06) and an aromatic carbon conjugated with a hydroxyl group (δ 163.4) indicated the presence of an enol in the coumarin moiety. Consequently, **3** was confirmed to be the enol form of methylated ω-hydroxyferulenol (Figure 4).

Compound **5** was obtained as an amorphous solid with the molecular formula C_25_H_32_O_4_ (*m/z* 397.2375 [M+H]^+^ calculated for C_25_H_33_O_4_, 297.2379; Figure S8) according to an HR-ESIMS analysis, which is consistent with that of **3**. The ^1^H NMR spectrum of **5** (Figure S9) was also similar to that of **3**; however, the ^13^C NMR spectra of both compounds differed in the low magnetic field region. Specifically, the chemical shift at δ 163.4 in the ^13^C NMR spectrum of **3** shifted to δ 177.4 in the corresponding spectrum of **5** (Figure S10), which suggests the presence of a ketone in the latter compound and reveals the occurrence of a keto–enol tautomerization between both compounds, as shown in Figure 4. Accordingly, compound **5** was determined to be methylated ω-hydroxyferulenol containing a keto moiety in the coumarin skeleton (Figure 4).

Compounds **4** and **6** were obtained as amorphous solids and their molecular formula was determined to be C_25_H_32_O_4_ via HR-ESIMS (**4**, *m/z* 403.2253 [M+Na]^+^ calculated for C_25_H_32_O_3_Na, 403.2249, and **6**, *m/z* 381.2431 [M+H]^+^ calculated for C_25_H_33_O_3_, 381.2430; Figures S11 and S12). The molecular weight of **4** and **6** was 16 units smaller than that of **3** and **5**, which suggests that the former compounds contain one hydroxyl group less than the latter. Hence, **4** and **6** were determined to be methylated ferulenol (Figure 4).

According to the chemical structure analyses, **1** and **2** formed keto–enol isomers in hydrogen donor solvents. Therefore, to avoid isomerization, the isolation and purification of these compounds were performed using aprotic solvents such as hexane, chloroform, and ethyl acetate. As mentioned in the Experimental Section, **1** and **2** afforded a single peak upon column chromatography and preparative HPLC purification, and were identified as ω-hydroxyferulenol (**1**) and ferulenol (**2**), respectively (Figure 4), on the basis of the comparison of their 1D NMR spectroscopy and HR-ESIMS analysis (Figures S1 and S2) with literature data [31].

### 2.3. Anti-Tube-Forming Activity of ω-Hydroxyferulenol (***1***) and Ferulenol (***2***)

**Figure 5:**
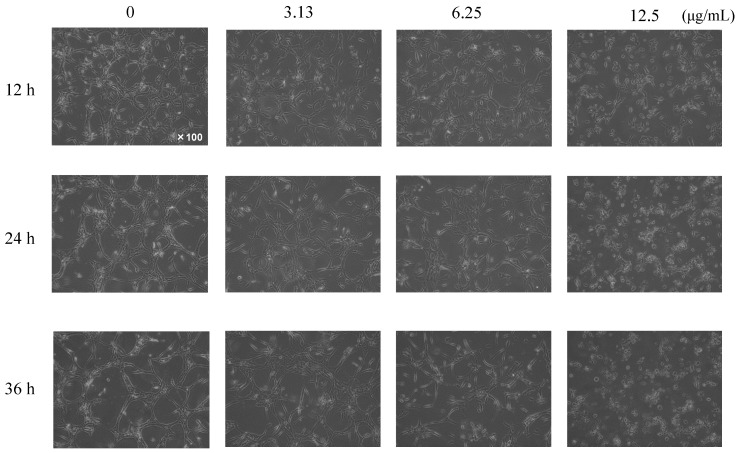
Inhibitory effects of ferulenol (**2**) on the tube formation of HUVECs. The compound showed a very strong antiangiogenic activity, inhibiting the formation of capillary networks and inducing cell fragmentation dose-dependently even at lower concentrations.

The effect of the isolated compounds ω-hydroxyferulenol (**1**) and ferulenol (**2**) on the angiogenesis of HUVECs was examined in vitro by performing a tube formation assay. HUVECs were treated with these compounds at concentrations of 3.31, 6.25, and 12.5 μg/mL for 12, 24, and 36 h. After the induction of the tube formation, the control endothelial cells formed a network of capillary-like tubes in the absence of **1** and **2**. Treatment with ω-hydroxyferulenol (**1**) at 12.5 μg/mL inhibited the formation of capillary networks, suppressing the elongation of HUVECs after 12 h and inducing cell fragmentation after 24 h (data not shown). Meanwhile, ferulenol (**2**) had a stronger antiangiogenic effect on tube-forming HUVECs, suppressing the elongation of HUVECs and inducing cell fragmentation even at a low concentration of 3.13 μg/mL (Figure 5).

### 2.4. Apoptosis Induction of Ferulenol (***2***)

**Figure 6:**
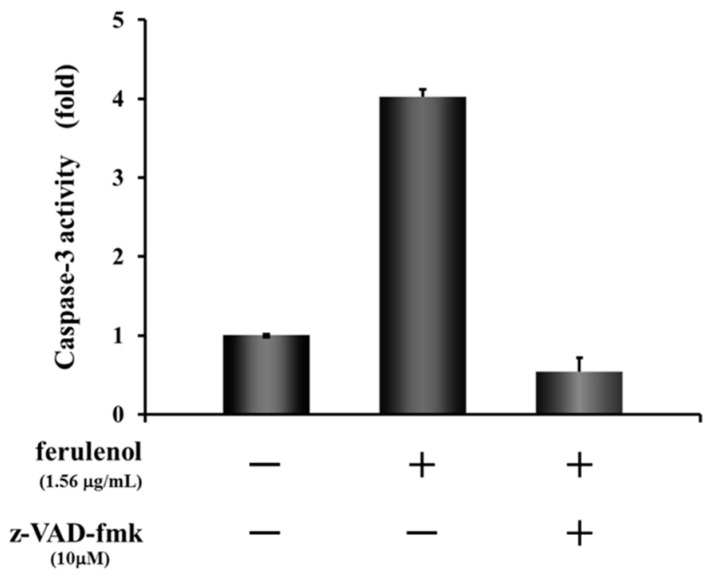
Effect of ferulenol (**2**) on apoptosis signal.

As mentioned above, although both compounds ω-hydroxyferulenol (**1**) and ferulenol (**2**) inhibited the tube formation of endothelial cells, **2** apparently showed much stronger antiangiogenic activity on HUVECs. To identify the molecular changes in tube-forming HUVECs that led to the cell fragmentation after treatment with ferulenol (**2**), the activation state of the apoptosis signal caspase-3/7 was analyzed. Treatment of HUVECs with ferulenol (**2**) at 1.56 μg/mL for 12 h activated caspase-3. Moreover, cotreatment with ferulenol (**2**) and z-VAD-fmk, which is an inhibitor of caspases, suppressed the caspase-3 activation (Figure 6). These results showed that ferulenol (**2**) activated the caspase signals, thereby inducing apoptosis and contributing to the inhibition of tube formation.

### 2.5. Identification of the Plant Origin of the Studied Propolis

**Figure 7:**
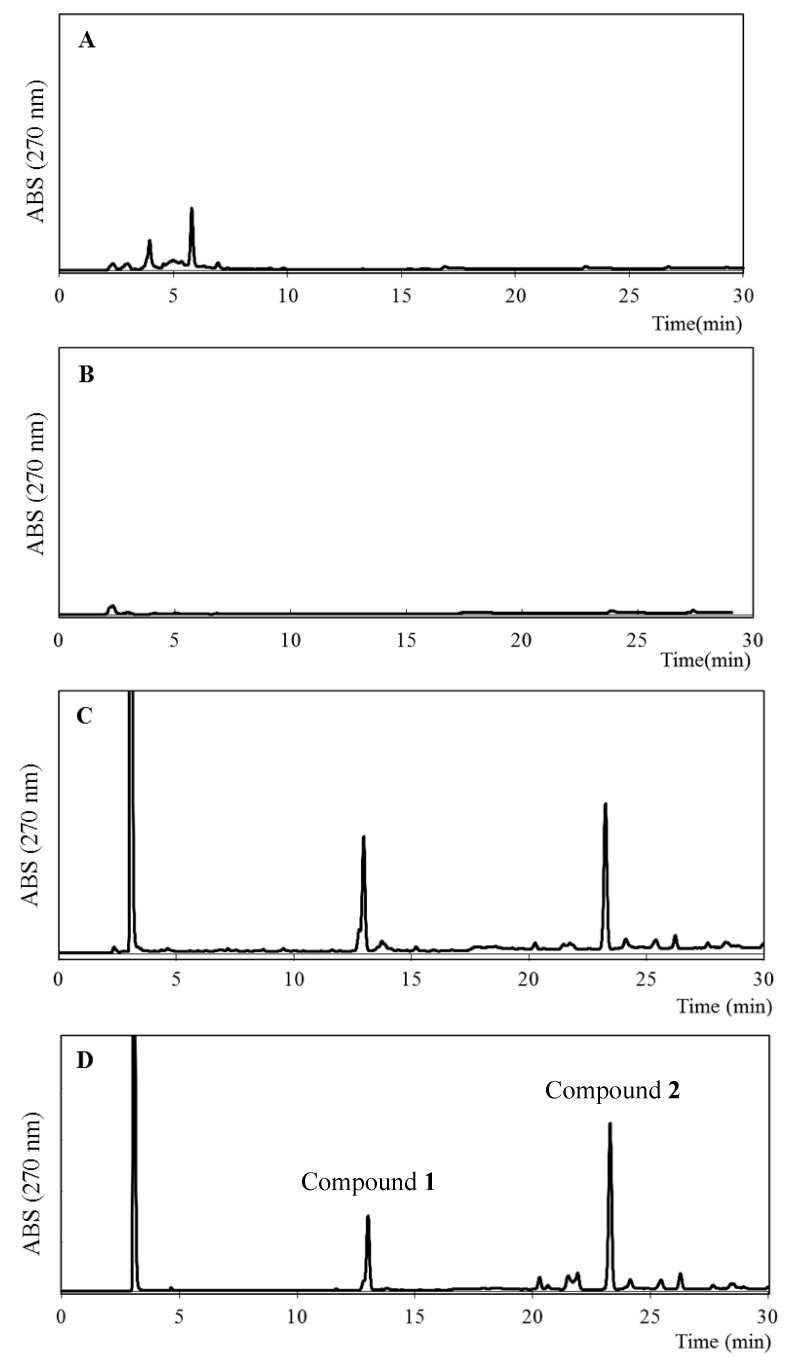
HPLC profiles of ethanol extracts of *I. viscosa* (**A**), *J. phoenicea* (**B**), *F. communis* (**C**), and EEAP1 (**D**).

The ethanol extracts of some of the plants grown around the bee farm in western Algeria, i.e., *Inula viscosa, Juniperus phoenicea*, and *Ferula communis*, were investigated using reverse-phase HPLC (RP-HPLC) coupled with HR-ESIMS analysis. As shown in Figure 7, the chromatogram of *F. communis* was similar to that of EEAP1, which suggests that *F. communis* might be one of the source plants of Algerian propolis.

## 3. Discussion

Although beekeeping is practiced in Algeria, the byproduct propolis has not been thoroughly studied yet. In this study, to investigate the potential utility of Algerian propolis, its main components were isolated and analyzed, leading to the identification of the active compounds **1** and **2**, whose antiangiogenic activity was examined. Furthermore, the plant origin of the propolis studied was identified.

Compounds **1** and **2** were isolated from EEPA1 and determined to be ω-hydroxyferulenol (**1**) and ferulenol (**2**) via NMR and HR-ESIMS analysis. It has been reported that these prenylated coumarins are active components of the latex of *Ferula communis* [31], which has been identified as the plant origin near the beehive box. Similar to our findings, a previously reported structure–relationship study revealed that ferulenol exhibits stronger antimycobacterial activity than that of hydroxyferulenol [32]. Another study reported that ferulenol shows cytotoxicity on liver FAO cells and B16F1 melanoma by inducing proapoptotic effects via a decrease in the mitochondrial membrane potential and mitochondrial respiratory rate [33]. Moreover, Bocca et al. reported the cytotoxic activity of ferulenol against various human tumor cell lines and its mechanism of action, which involves the stimulation of tubulin polymerization and the inhibition of the binding of colchicine to tubulin [34]. As far as we know, reports on the biological activities of ω-hydroxyferulenol and ferulenol are very scarce.

In this biological study, the antiangiogenic effect of ω-hydroxyferulenol (**1**) and ferulenol (**2**) on tube formation and cell fragmentation was demonstrated in vitro. Ferulenol (**2**) exhibited antiangiogenic activity even at a low concentration of 3.13 μg/mL. These results suggest that ferulenol (**2**), which has a prenylated moiety, shows stronger activity at lower concentrations when compared with the hydroxy-prenylated compound ω-hydroxyferulenol (**1**).

The molecular changes in fragmented HUVECs after treatment with ferulenol (**2**) were examined and the activation of caspase-3 at the end of the apoptotic signal was confirmed. Treatment with ferulenol (**2**) at 1.56 μg/mL for 12 h activated caspase-3 and this activation was suppressed by adding the inhibitor z-VAD-fmk. Hence, the caspase activation experiments revealed that cell fragmentation was associated with apoptosis. These results indicate that induction of apoptosis is a critical mechanism of angiogenesis suppression by ferulenol (**2**). Although we were able to show that ferulenol (**2**) exhibited antiangiogenesis by inhibiting tube formation and inducing apoptosis in HUVECs in vitro, it remains to be seen whether such activity can also be observed in vivo. Nonetheless, this is the first report demonstrating the antiangiogenic activity of ferulenol on HUVECs, which proceeds via induction of apoptosis by inhibiting the caspase signals. Thus, Algerian propolis may have future applications in the prevention and treatment of angiogenesis-related diseases.

## 4. Materials and Methods

### 4.1. Materials and Chemicals

Medium 199, medium MCDB-104, and fetal bovine serum (FBS) were purchased from Sigma (St. Louis, MO, USA), Nihon Pharmaceutical (Tokyo, Japan), and Moregate (Brisbane, Australia), respectively. Cellgen was obtained from Koken (Tokyo, Japan). The epidermal growth factor (EGF) and human basic fibroblast growth factor (FGF; recombinant) were purchased from BD Biosciences (Bedford, MA, USA) and the Wako Pure Chemical Corporation (Osaka, Japan), respectively.

### 4.2. Equipment for the Isolation and Structure Analysis

One-dimensional (^1^H and ^13^C) and two-dimensional NMR spectra were measured on a Bruker AVANCE III 400 instrument (Bruker BioSpin, Billerica, MA, USA). Chemical shifts (*δ*) were reported in parts per million and coupling constants (*J*) were reported in hertz. The chemical shifts in the ^1^H and ^13^C NMR spectra were corrected using the residual solvent signals. HR-ESIMS was performed using an Accela LC system (Thermo Fisher Scientific, Waltham, MA, USA) equipped with a quadrupole mass spectrometer (Q-Exactive; Thermo Fisher Scientific). RP-HPLC analyses were conducted using a PU-2086 Plus intelligent prep pump (Jasco Co., Inc., Tokyo, Japan) with the UV-970 UV/VIS detector (Jasco Co., Inc.), a Shiseido Capcell Pak C18 UG120 (4.6 × 250 mm) column for analysis, and a Shiseido Capcell Pak C18 U120 (20 × 250 mm) column for preparative purposes. Preparative normal-phase HPLC was performed using the same pump and detector with a Nomura Chemical (Aichi, Japan) Develosil 60-5 (7.5 × 250 mm) column.

### 4.3. Propolis Samples

Propolis samples were collected from beehives located at different regions in western Algeria, i.e., Tiaret (Samples 1 and 2), Tlemcen (Sample 3), and Sidi bel Abbés (Sample 4), in March–May 2010. Additionally, the sampling collection was conducted under controlled conditions (temperature, light, etc.), and professional beekeepers who did not use any antibiotics or other chemicals for treating the beehives were selected. Samples were stored at −20 °C in our laboratory.

### 4.4. Screening of the Four Algerian Propolis Samples and Isolation of Active Compounds (***1*** and ***2***)

The four propolis samples collected in Algeria were dried and extracted with ethanol at room temperature for 24 h with stirring. After the evaporation of ethanol, the extract was dissolved in dimethylsulfoxide to investigate the inhibition activity of tube formation at a concentration of 50 μg/mL.

Algerian propolis (Sample 1; 10.18 g), which showed the strongest activity for the inhibition of tube formation, was extracted with 500 mL of ethanol for 24 h with stirring and the subsequent evaporation of ethanol afforded EEAP1. The EEAP1 extract (9.85 g) was dissolved in hexane, placed on top of a silica gel column (600 × 45 mm, i.d.), and eluted with a gradient of *n*-hexane/EtOAc and MeOH to produce 11 fractions. After the elution profile analysis, Fr. 4 contained the main compounds of the propolis.

After silica gel column chromatography, Fr. 4 (128.8 mg) of EEAP1 was dissolved in 0.5 mL of acetone and 1 mL of TMSCHN_2_ was added. After incubation at room temperature for 15 h, the solvent was evaporated to afford methylated Fr. 4 (147.2 mg), which was then subjected to RP-HPLC by eluting with a mobile phase composed of 32% (*v/v*) acetonitrile in H_2_O containing 0.1% trifluoroacetic acid to produce **3** (3.1 mg), **4** (1.9 mg), **5** (4.6 mg), and **6** (2.6 mg).

Apart from methylation, Fr. 4 (111 mg) was further chromatographed using a silica gel column (600 × 25 mm, i.d.) and eluted with a gradient of *n*-hexane/chloroform and acetone. Then, Frs. 4-4 and 4-5 were subjected to normal-phase HPLC and eluted for 60 min with a mobile phase composed of a gradient system of hexane and ethyl acetate (100:0–0:100) to produce pure compounds **1** (2.7 mg) and **2** (3.2 mg).

### 4.5. Cell Culture

HUVECs were isolated from the human umbilical cord and cultured in a HUVEC growth medium (MCDB-104 medium supplemented with 10% FBS, 10 ng/mL of EGF, 100 μg/mL of heparin, and 100 ng/mL of the endothelial growth factor). Incubation was conducted at 37 °C under a humidified 95%/5% (*v/v*) mixture of air and CO_2_. The cells were seeded on plates coated with 0.1% gelatin and allowed to grow to sub-confluence before experimental treatment.

### 4.6. Tube Formation Assay

Capillary tube-like structures formed by HUVECs in collagen gel were prepared following a previously described procedure with slight modifications [21]. Collagen gels were made using Cellgen (type I collagen). Collagen solution (200 μL, 0.21% in Medium-199) was poured into the wells of a 24-well plate and the plates were incubated at 37 °C for 30 min to solidify the gels. HUVECs (6.0 × 10^4^ cells/cm^2^) in MCDB-104 medium with 0.5% FBS were seeded onto the collagen-coated wells and left at 37 °C under 5% CO_2_ in an incubator for 1 h to allow them to attach to the collagen gel. After removing the medium, 150 μL of the collagen solution was overlaid and subjected to gelation as described above. Subsequently, 650 μL of MCDB-104 with 0.5% FBS supplemented with 10 ng/mL bFGF, 8 nM/mL of PMA (Phorbol 12-myristate 13-acetate), and various concentrations of samples were added to the wells and incubated for up to 36 h. The resulting web-like capillary structure was observed under a microscope with 100X magnification and captured with an Olympus C-4040ZOOM digital camera (Olympus Co., Tokyo, Japan).

### 4.7. Caspase-Glo 3/7 Assay

HUVECs (5.0 × 10^5^ cells/well) in MCDB-104 medium were seeded onto collagen-coated 96-well white-plate wells and left at 37 °C under 5% CO_2_ in an incubator for 1 h to allow them to attach to the collagen gel. After removing the medium, 100 μL of MCDB-104 with 0.5% FBS supplemented with 10 ng/mL bFGF, 8 nM/mL of PMA (Phorbol 12-myristate 13-acetate), and 3.31 μg/mL of ferulenol (**2**) with/without z-VAD-fmk was added to the wells and incubated for up to 12 h. After incubation, 80 μL of the medium was removed and 20 μL of Caspase-Glo 3/7 assay solution was both added and incubated for 1 h at room temperature. The chemiluminescence was recorded using Soft Max Pro.

### 4.8. Sampling of the Resin of Ferula Communis

The resin of *Ferula communis*, grown naturally near an apiary, was collected from the Sfisef province of Sidi bel Abbés, located in western Algeria (35°14′ N/0°14′ W), in September 2012.

### 4.9. Statistical Analysis

Experimental values are expressed as the mean of triplicate experiments ± standard deviation.

## 5. Conclusions

In this study, the antiangiogenic activity of the ethanol extract of four propolis samples (Samples 1–4) collected in western Algeria was investigated. To identify the active components in the ethanol extract exhibiting the strongest activity (EEAP1), repeated column chromatography and preparative HPLC were performed, which allowed for identifying the major active components as ω-hydroxyferulenol (**1**) and ferulenol (**2**). These compounds exhibited an antiangiogenesis effect in vitro on tube-forming HUVECs. Additionally, the plant origin of the studied propolis was determined to be most likely the resin of *F. communis* on the basis of a comparative analysis. This result indicates that Algerian propolis may have future applications in the prevention and treatment of angiogenesis-related diseases.

## Figures and Tables

**Table 1:** Collection areas and properties of the collected propolis samples.

Sample No.	Voucher Specimen	Area	Color and Texture
1	TIA-1	Tiaret	Dark brown and rigid
2	TIA-2	Tiaret	Dark brown and rigid
3	NED-TL	Tlemcen	Dark brown and waxy
4	SFS-SBA	Sidi bel Abbés	Brown, waxy, and sticky

## Data Availability

The data presented in this study are available upon request from the corresponding author.

## Supplementary Materials

The following are available online, Figure S1. HR-ESIMS spectrum of **1**, Figure S2. HR-ESIMS spectrum of **2**, Figure S3. HR-ESIMS spectrum of **3**; Figure S4. 1H NMR spectrum of **3** (400 MHz, acetone-d6); Figure S5. 13C NMR spectrum of **3** (100 MHz, acetone-d6); Figure S6. HSQC spectrum of **3** (acetone-d6); Figure S7. HMBC spectrum of **3** (acetone-d6); Figure S8. HR-ESIMS spectrum of **5**; Figure S9. 1H NMR spectrum of **5** (400 MHz, acetone-d6); Figure S10. 13C NMR spectrum of **5** (100 MHz, acetone-d6); Figure S11. HR-ESIMS spectrum of **4**; Figure S12. HR-ESIMS spectrum of **6**.

## Sample Availability

Samples are available from the authors upon reasonable request.

## Abbreviations

EEAP	ethanol extracts of Algerian propolis
HPLC	high-performance liquid chromatography
HUVECs	human umbilical vein endothelial cells
NMR	nuclear magnetic resonance
HR-ESIMS	high-resolution electrospray ionization mass spectroscopy
TMS	trimethylsilyl
