# Supplementary material for: Composition of Algerian Propolis, Plant Origin, and Its Antiangiogenic Activity In Vitro
Source: Molecules. 2021 Oct 28;26(21):6510. doi: 10.3390/molecules26216510 (PMC8587774; doi:10.3390/molecules26216510)
Supplement: Supplementary file 1 [file molecules-26-06510-s001.zip › molecules-1421489-supplementary.pdf]

# Supplemental material

# Composition of Algerian Propolis, Plant Origin and its Antiangiogenic Activity In Vitro

Takahiro Hosoya [1,2], Ikumi Tsuchiya [1], Toshiro Ohta [1], Mokhtar Benhanifia [3] and Shigenori Kumazawa [1,*]

[1] Department of Food and Nutritional Sciences, University of Shizuoka, 52-1 Yada, Suruga-ku, Shizuoka 422-8526, Japan

[2] Department of Nutrition and Health Sciences, Toyo University, 1-1-1, Izumino, Itakura-machi, Ora-gun, Gunma 374-0193, Japan

[3] Department of Agricultural Science, Faculty of Natural and Life Sciences, University Mustapha Stambouli of Mascara, 29000 Algeria

* Correspondence: kumazawa@u-shizuoka-ken.ac.jp; Tel.: +81-54-264-5523

Contents

**Figure S1.** HR-ESIMS spectrum of **1**. ……………………………………………….3

**Figure S2.** HR-ESIMS spectrum of **2**. ……………………………………………….3

**Figure S3.** HR-ESIMS spectrum of **3**. ……………………………………………….4

**Figure S4.** $^{1}$H NMR spectrum of **3** (400 MHz, acetone-$d_6$). ……………………………4

**Figure S5.** $^{13}$C NMR spectrum of **3** (100 MHz, acetone-$d_6$). ……………………………5

**Figure S6.** HSQC spectrum of **3** (acetone-$d_6$). ………………………………………….5

**Figure S7.** HMBC spectrum of **3** (acetone-$d_6$). ………………………………………….6

**Figure S8.** HR-ESIMS spectrum of **5**. ………………………………………………..7

**Figure S9.** $^{1}$H NMR spectrum of **5** (400 MHz, acetone-$d_6$). ……………………………7

**Figure S10.** $^{13}$C NMR spectrum of **5** (100 MHz, acetone-$d_6$). ……………….……….8

**Figure S11.** HR-ESIMS spectrum of **4**. ……………………….……………………...9

**Figure S12.** HR-ESIMS spectrum of **6**. ……………………….……………………...9

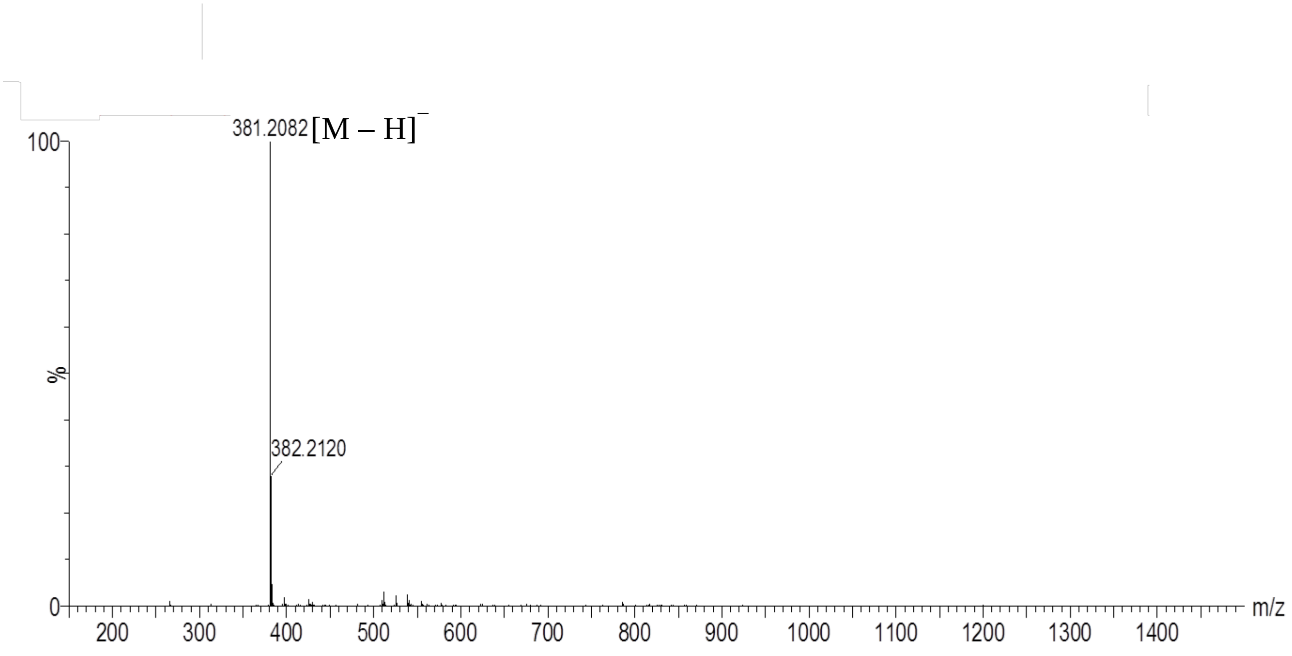


**Figure S1.** HR-ESIMS spectrum of **1**.

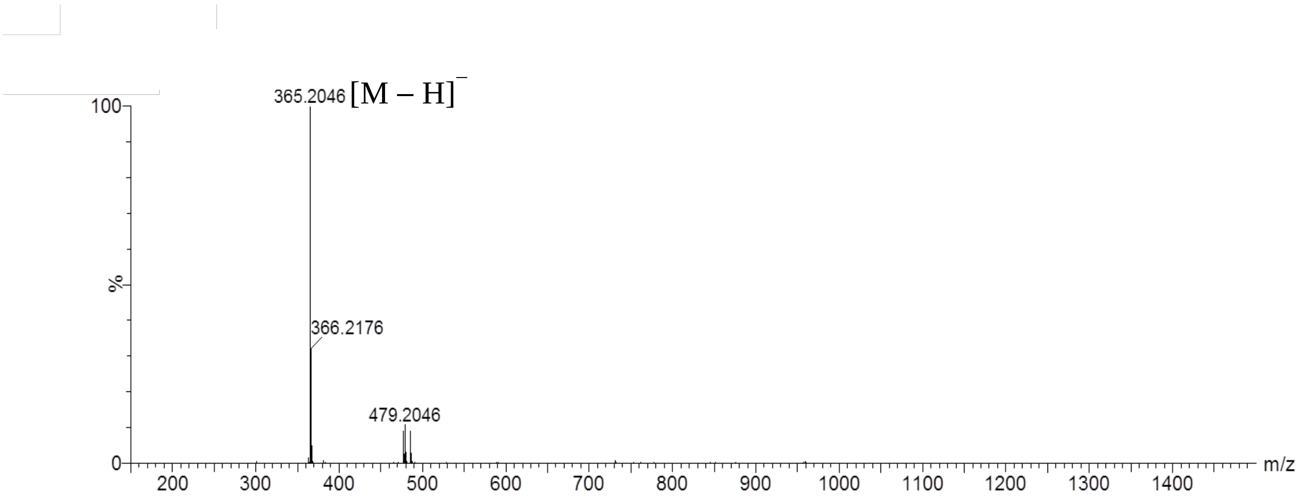


**Figure S2.** HR-ESIMS spectrum of **2**.

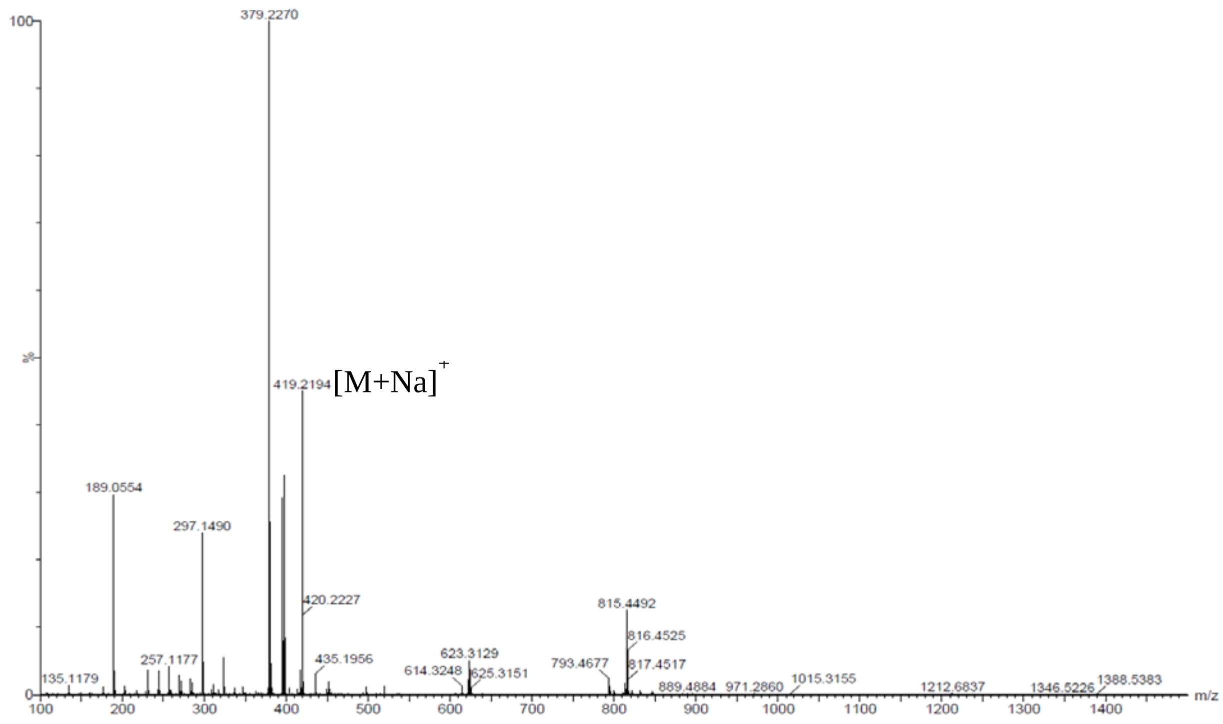


**Figure S3.** HR-ESIMS spectrum of **3**.

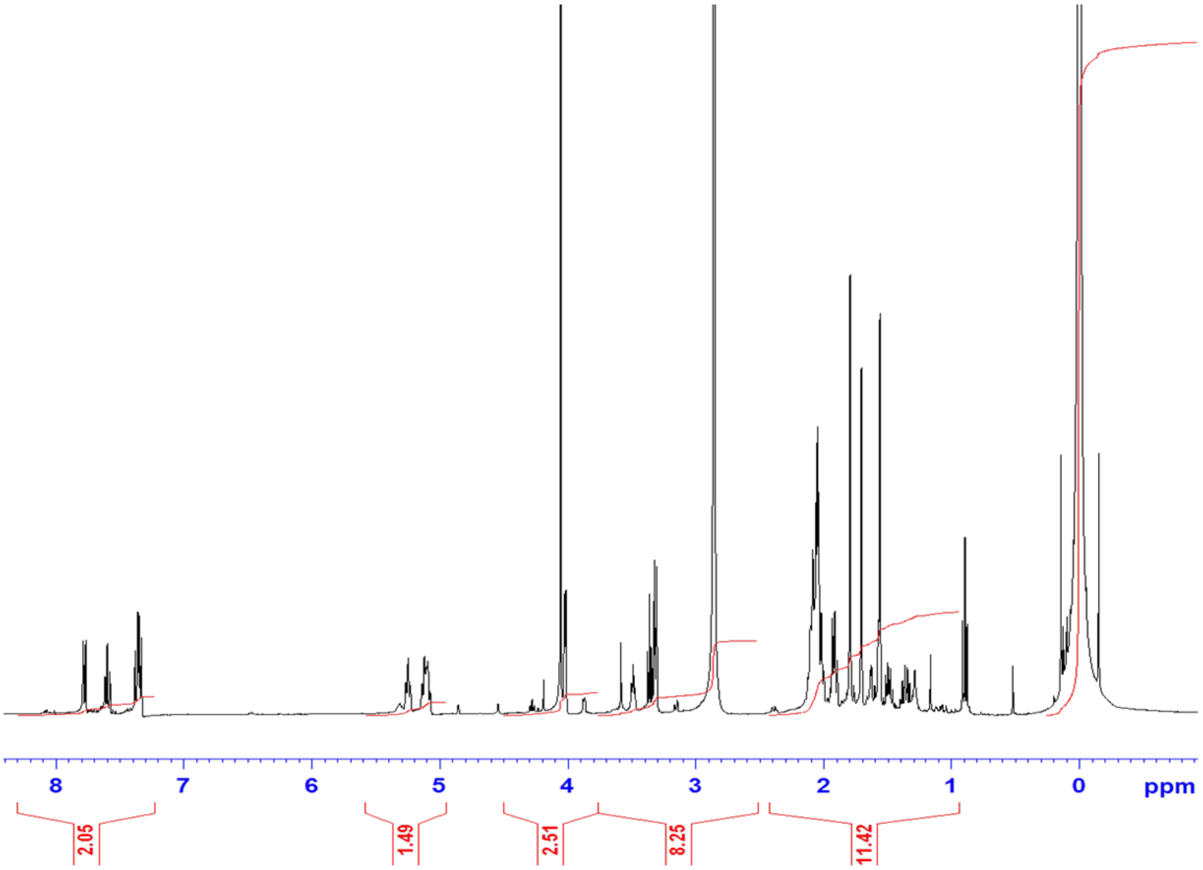


**Figure S4.** $^{1}$H NMR spectrum of **3** (400 MHz, acetone-$d_6$).

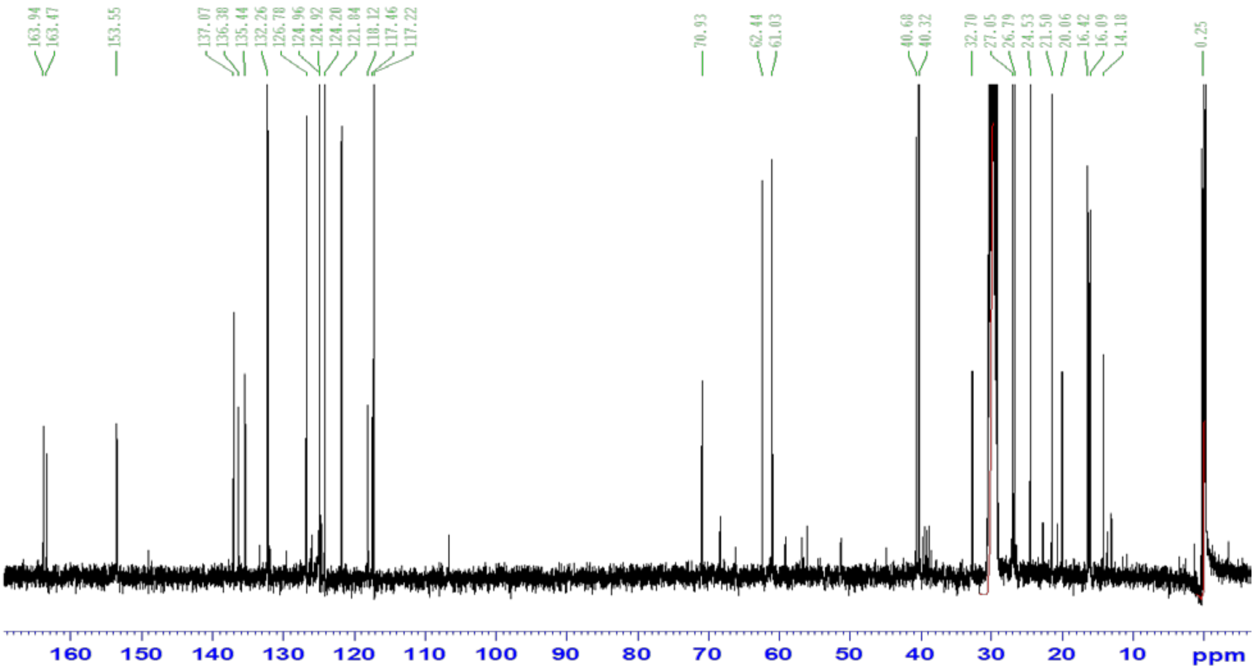


**Figure S5.** $^{13}$C NMR spectrum of **3** (100 MHz, acetone-$d_6$).

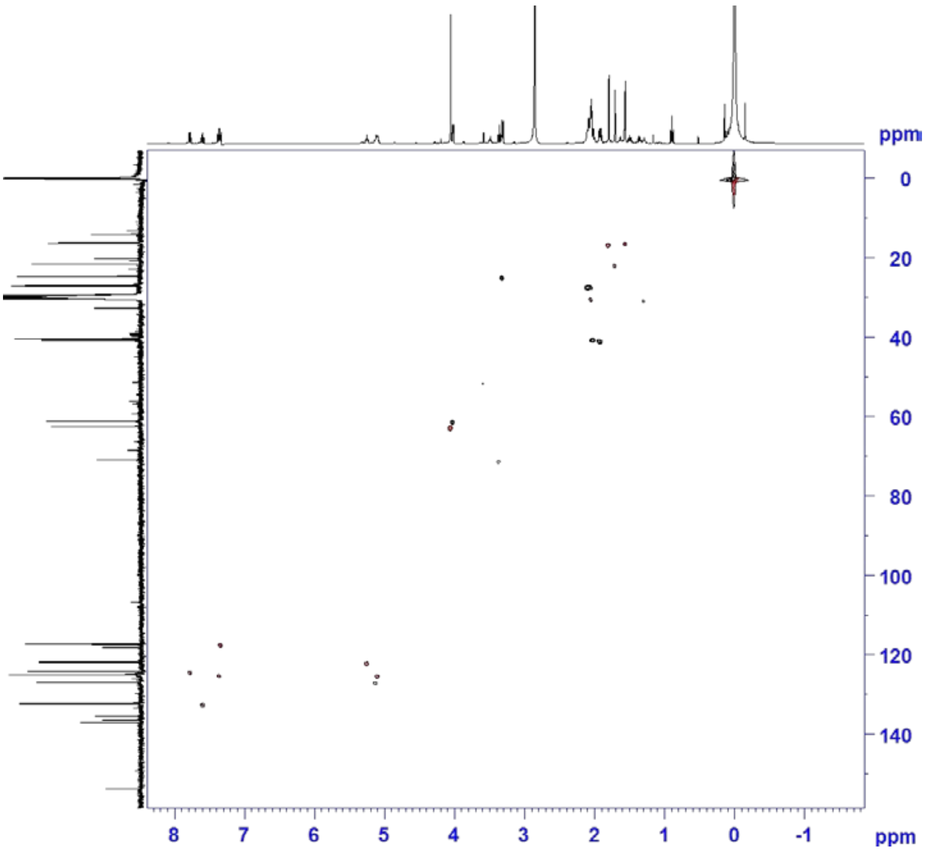


**Figure S6.** HSQC spectrum of **3** (acetone-$d_6$).

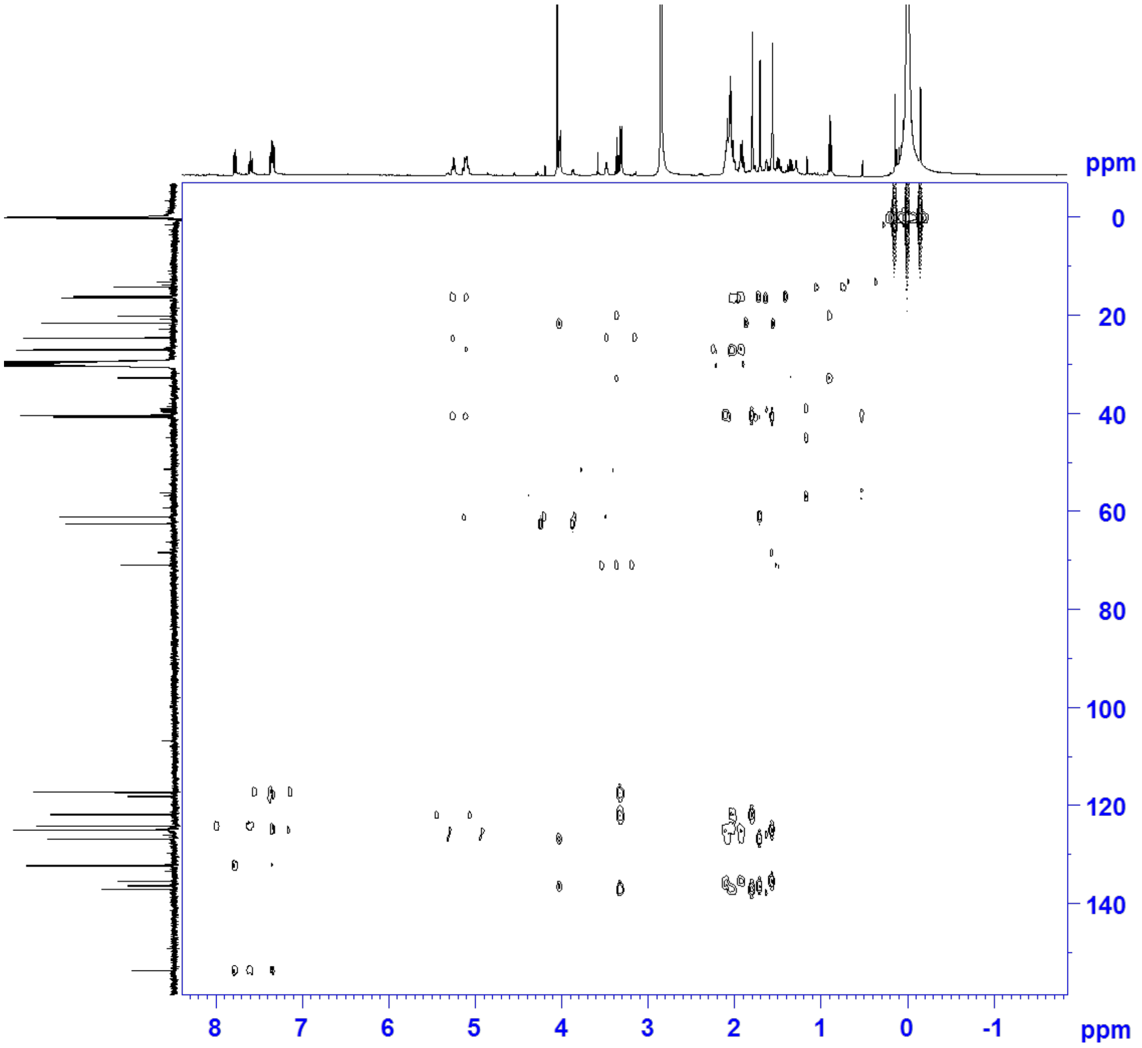


**Figure S7.** HMBC spectrum of **3** (acetone-$d_6$).

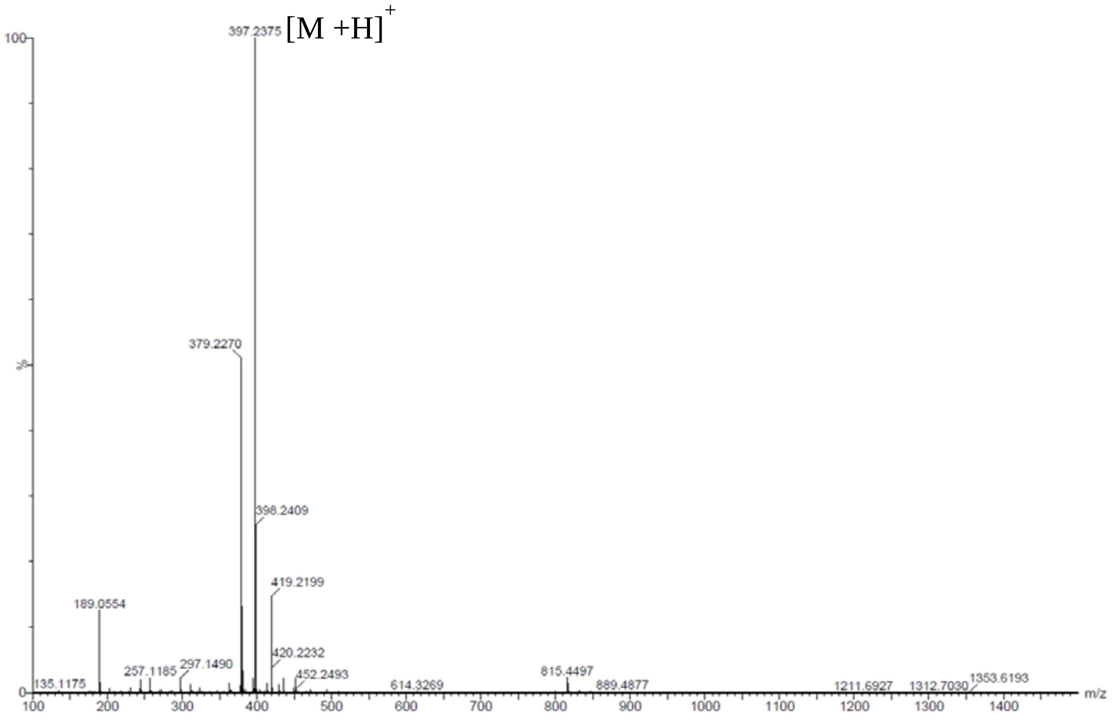


**Figure S8.** HR-ESIMS spectrum of **5**.

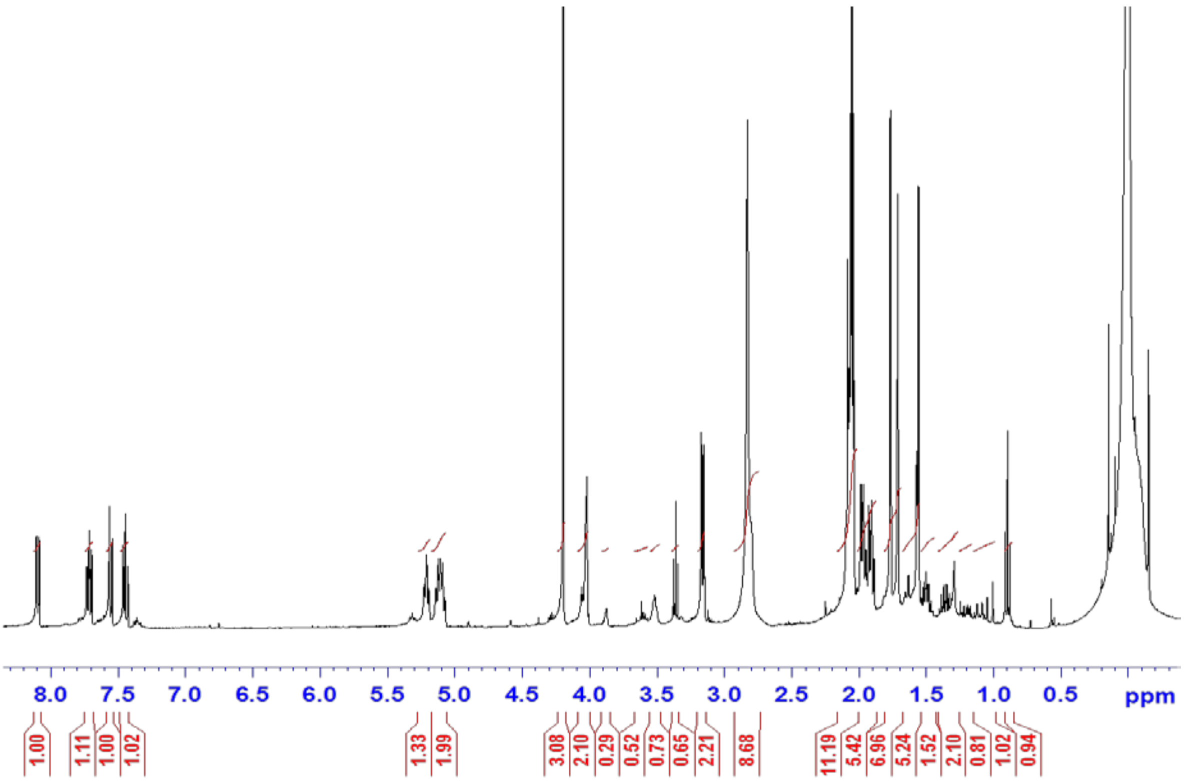


**Figure S9.** $^{1}$H NMR spectrum of **5** (400 MHz, acetone-$d_6$).

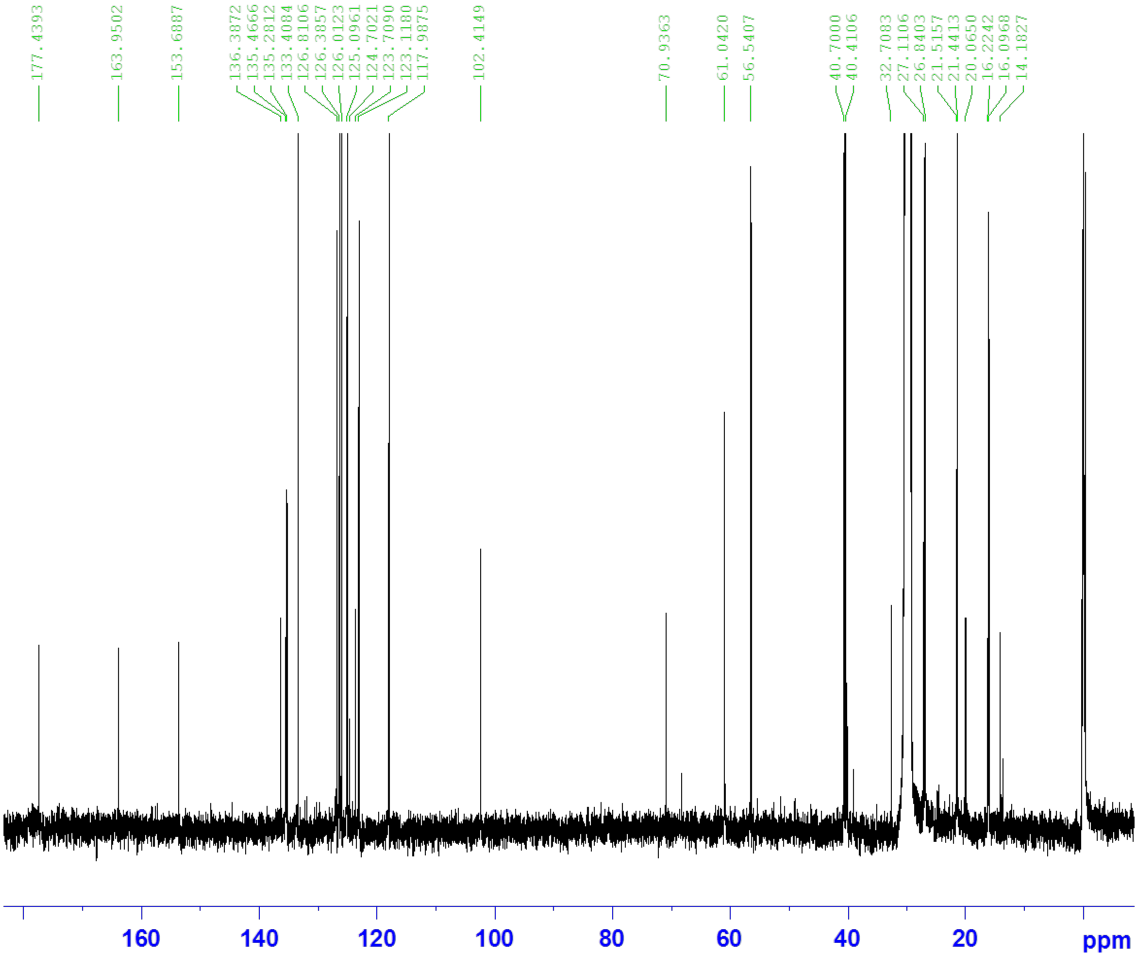


**Figure S10.** $^{13}C$ NMR spectrum of **5** (100 MHz, acetone-$d_6$).

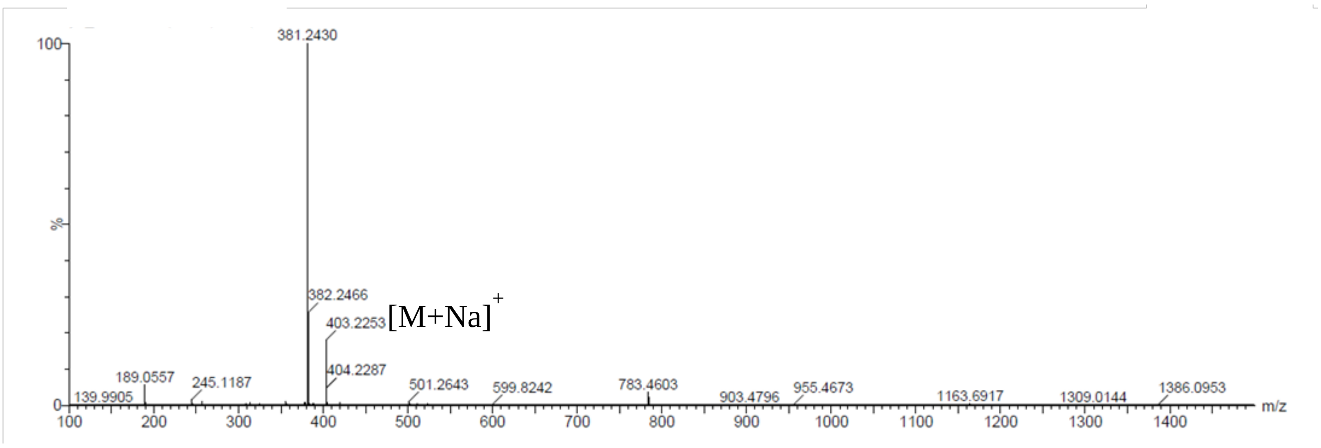


**Figure S11.** HR-ESIMS spectrum of **4**.

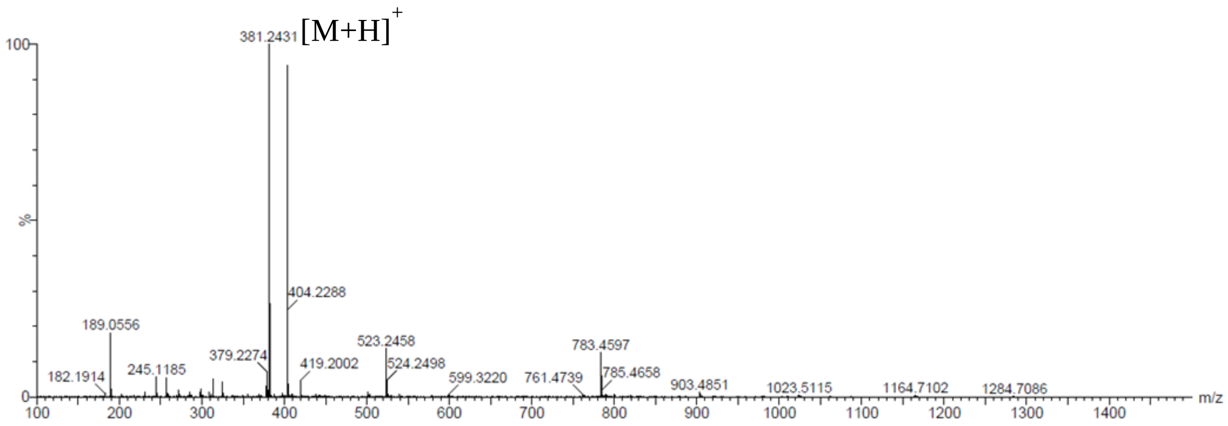


**Figure S12.** HR-ESIMS spectrum of **6**.
